# Case report: Chylopericardium secondary to dialysis catheter related jugular venous thrombosis in two dogs receiving long-term hemodialysis

**DOI:** 10.3389/fvets.2024.1386710

**Published:** 2024-05-20

**Authors:** Dennis J. Woerde, Carrie A. Palm, Laetitia M. Duler, Larry D. Cowgill, Marisa K. Ames, William T. N. Culp

**Affiliations:** ^1^William R Pritchard Veterinary Medical Teaching Hospital, University of California, Davis, Davis, CA, United States; ^2^Department of Veterinary Medicine and Epidemiology, University of California, Davis, Davis, CA, United States; ^3^Department of Surgical & Radiological Sciences, University of California, Davis, Davis, CA, United States

**Keywords:** chylous, dialysis, tissue plasminogen activator, canine, pericardial, clopidogrel

## Abstract

Chylopericardium is a rare entity in veterinary medicine. In this report we document the development of chylopericardium in two dogs undergoing chronic hemodialysis. An 11-year-old female spayed Labrador retriever (Case 1) presented with acute coughing and lethargy 2 months following initial dialysis catheter placement and initiation of dialysis therapy for severe azotemia. Echocardiography demonstrated severe pericardial effusion and cardiac tamponade. Pericardial fluid analysis was consistent with chylous effusion. The dog underwent a subtotal pericardiectomy with thoracic duct ligation, and a PleuralPort™ was placed. The patient continued to receive outpatient hemodialysis therapy after pericardiectomy for several months until she died acutely at home. A 4-year-old male neutered Doberman (Case 2) was being treated for 2 months with outpatient hemodialysis for management of chronic kidney disease. On presentation for the 17th hemodialysis treatment, the patient had increased respiratory rate. Echocardiography demonstrated pleural and pericardial effusions, and fluid analysis in both cavities was consistent with chylous effusion. Use of tissue plasminogen activator (TPA), clot removal and replacement of the catheter was attempted; however pleural and pericardial effusion continued. The patient was euthanized after 25 hemodialysis sessions as owners elected not to pursue more procedures. In both cases, the cause of the chylopericardium was suspected to be secondary to catheter-associated thrombosis and/or stenosis based on multiple imaging modalities. Despite use of rivaroxaban and clopidogrel concurrently in each case, the chylous effusion persisted. This case report describes clinical details of a rare complication of long-term indwelling dialysis catheters in two dogs.

## Introduction

Chylopericardium is a rare medical condition characterized by accumulation of chylous fluid within the pericardial cavity (1, 2). Causes in humans include cardiothoracic trauma, mediastinal neoplasia, radiation therapy, jugular or subclavian vein thrombosis/stenosis, congenital lymphatic malformations, tuberculosis and other causes of elevated central venous pressure (1–6). No obvious cause is identified in ~50% of humans with chylopericardium (1, 2).

Only two previous cases of chylopericardium in dogs are reported in the literature (4, 7), none of which resulted from indwelling catheters with subsequent venous thrombosis and/or stenosis. As initiation of extracorporeal therapies become more common in veterinary medicine, development of catheter-induced chylopericardium is an important consequence that clinicians should be cognizant of. This report details development of chylopericardium in two dogs with long term jugular venous dialysis catheters receiving chronic dialysis.

## Case report

### Case 1

An 11-year-old female spayed 28 kg Labrador retriever was referred for severe azotemia. On initial evaluation the dog was obtunded with a body condition score of 3/9. Initial bloodwork revealed a moderate normocytic, normochromic non-regenerative anemia of 20% (RI 40%−55%). Marked azotemia was present, with a serum creatinine of 13 mg/dl (RI: 0.8–1.5), BUN of 168 mg/dl (RI: 11–33) and a metabolic acidosis (bicarbonate 12 mmol/L, RI: 20–29), marked hyperphosphatemia (14.4 mg/dl, RI: 2.6–5.2) and moderate hypoalbuminemia (2.9 g/dl, RI: 3.4–4.3). A urine protein:creatinine ratio was mildly elevated at 1.2 (RI < 0.5). Systolic blood pressure was elevated at 180–190 mmHg. Abdominal ultrasound revealed renal changes consistent with severe chronic kidney disease (CKD). Thoracic radiographs revealed no intrathoracic abnormalities. The patient was diagnosed with oligoanuric IRIS stage 4 CKD based on diagnostic work up and lack of urine production. Hemodialysis was pursued.

**Figure 1:**
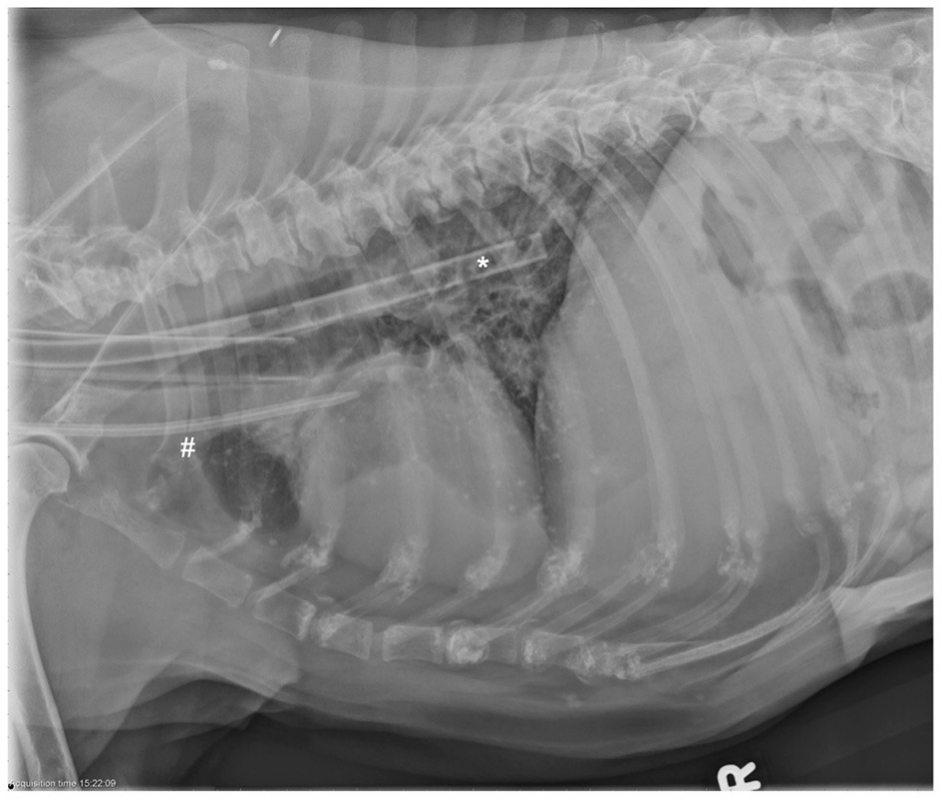
Right lateral thoracic radiographs confirming termination of the jugular catheter (labeled #) in the region of the cranial vena cava and right atrium. A radiopaque esophageal feeding tube (labeled *) is also present, terminating at the level of the lower esophageal sphincter.

A 14-French (Fr) × 24 cm double lumen silicone jugular catheter (MILA International, Inc, Florence, Kentucky) was placed aseptically into the right jugular vein without complication. A 30 Fr × 55 cm esophageal feeding tube (E-tube; MILA International, Inc, Florence, Kentucky) was placed concurrently without complication. Thoracic radiographs confirmed appropriate dialysis catheter positioning (Figure 1).

The patient was initially hospitalized for 10 days and received five hemodialysis sessions using systemic unfractionated heparin (UFH) as anticoagulation to achieve an ACT of 180–220 seconds during therapy. The patient was discharged and returned for thrice weekly outpatient hemodialysis therapy, receiving the following medications via the E-tube: amlodipine 0.2 mg/kg SID, maropitant 2 mg/kg SID, aluminum hydroxide 85 mg/kg/day divided with each feeding, sodium bicarbonate 68 mg/kg BID, taurine 500 mg SID, carnitine 500 mg SID and clopidogrel 1.5 mg/kg SID. The catheter was heparin-locked (100–110 units/kg/ml UFH) between hemodialysis treatments. Darbepoetin (Aranesp; Amgen, Thousand Oaks, CA) was also given subcutaneously at 1 μg/kg weekly.

**Figure 2:**
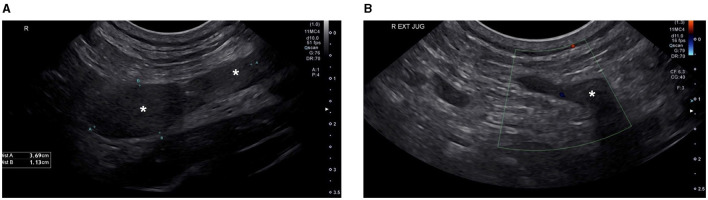
Cervical ultrasound ultasound images. Ultrasound image **(A)** shows an extensive echogenic thrombus (labeled *) filling the majority of the lumen of the right external jugular vein. Ultrasound image **(B)** shows the absence of Color Doppler flow signal in the right external jugular vein at the level of the thrombus, confirming obstruction of the vessel by the intraluminal thrombus.

Two weeks into outpatient hemodialysis management (11 total treatments) the patient presented for scheduled hemodialysis therapy. Facial and right forelimb swelling was observed. An echocardiogram revealed no significant abnormalities, however based on the clinical presentation, catheter associated thrombosis was suspected. Repeat serum biochemistry revealed static azotemia from initial presentation and severe hyperkalemia of 6.7 mmol/L (RI: 3.6–4.8). Hemodialysis was performed as scheduled. Furosemide at 0.5 mg/kg BID and rivaroxaban at 0.6 mg/kg SID via the E-tube were prescribed. All previously dispensed medications were continued. The patient was discharged and returned 3 days later for scheduled hemodialysis. The patient's facial and right forelimb swelling had progressed. A cervical ultrasound revealed an extensive, echogenic thrombus filling the majority of the right jugular vein lumen and variably expanding the right external jugular vein along its length (Figure 2A). The thrombus obstructed venous blood flow, with lack of ultrasonographic Doppler flow (Figure 2B). Caudally, the thrombus traversed the right brachiocephalic vein with extension into the right axillary vein, incompletely filling the lumen. At this juncture rivaroxaban was increased to 0.8 mg/kg BID and clopidogrel increased to 2.3 mg/kg SID via the E-tube.

**Figure 3:**
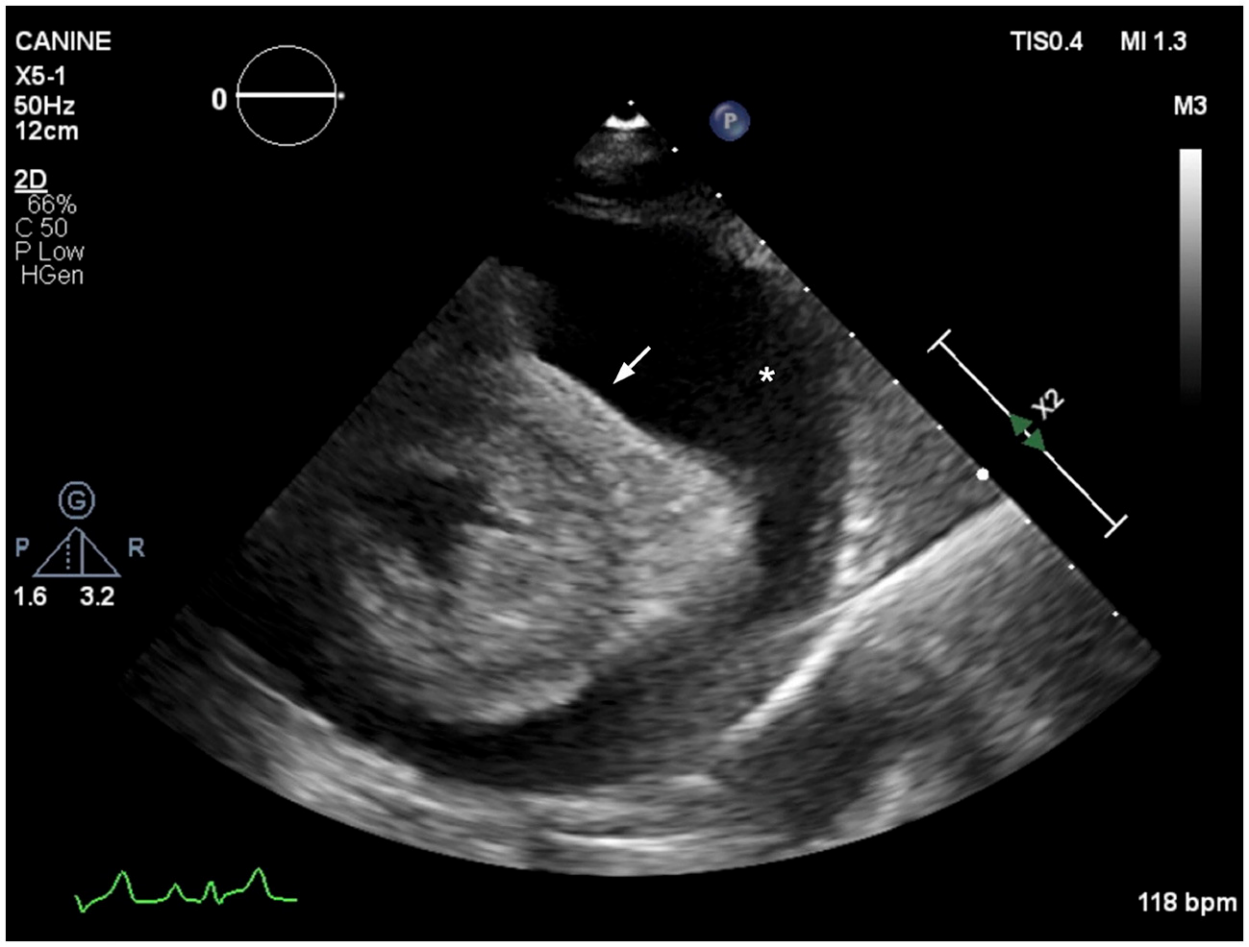
Echocardiographic image (right parasternal short axis view) showing severe anechoic pericardial effusion (labeled as *). The right ventricular cavity is obliterated during diastole (arrow), consistent with cardiac tamponade.

Over the following 4 weeks the patient's facial and right forelimb swelling steadily improved. The patient continued to receive hemodialysis therapy thrice weekly. At the 26th hemodialysis session (2 months into outpatient hemodialysis) the patient was coughing, with increased respiratory rate and muffled heart sounds. Thoracic radiographs revealed pleural effusion and cardiac silhouette enlargement. An echocardiogram revealed severe pericardial effusion with evidence of cardiac tamponade (Figure 3) and mild-to-moderate pleural effusion. No echocardiographic evidence of cardiac neoplasia or intracardiac thrombosis was present. Recheck cervical ultrasound revealed improved though persistent right jugular vein thrombosis. A pericardiocentesis was performed using a 14 gauge (G) × 5.25 inch BD Angiocath™ catheter (BD, Edison, NJ) between the 4th and 6th right intercostal space after 1 ml of 2% lidocaine local block, removing 245 ml of pink opaque effusion. Subsequent thoracocentesis was performed using an additional 14G × 5.25 inch BD Angiocath™ catheter (BD, Edison, NJ) between the 7th and the 9th ribs and 411 ml of straw-colored pleural fluid was removed. Pericardial effusion analysis was consistent with a chylous effusion, with triglyceride content 655 mg/dl (serum triglycerides; 54 mg/dl) and positive Sudan III staining. Pleural effusion analysis was consistent with a modified transudate, with total protein 3.0 g/dl and total nucleated cells 940/μl. The pleural fluid was not chylous in nature, with triglyceride content of 80 mg/dl (serum triglycerides; 54 mg/dl) and was suspected to be secondary to cardiac tamponade. Culture of fluid samples was not performed. The patient was discharged the following day on previously prescribed medications.

The patient returned 48 h later for a scheduled hemodialysis treatment. On presentation the patient was tachycardic (heart rate: 180 bpm) with muffled heart sounds and weak femoral pulses. A point-of-care thoracic ultrasound scan revealed recurrence of pericardial effusion. Pericardiocentesis was performed as described previously, removing 500 ml of pink opaque fluid. Clinicopathologic evaluation was not repeated. The patient was hospitalized and hemodialysis was performed the following day. Several hours after hemodialysis, a repeat echocardiogram revealed recurrent pericardial effusion with no overt evidence of constrictive physiology. Another pericardiocentesis was performed, with an additional 350 ml of opaque fluid removed. To further investigate the effusion, a thoracic computed tomography (CT) scan was performed revealing cranial mediastinal and proximal thoracic limb edema and/or lymphatic distension with enlarged regional lymph nodes. Thrombosis was present alongside the dialysis catheter extending into the cranial vena cava. A concurrent lymphangiogram was attempted but was unsuccessful.

Aside from venous thrombosis, no other definitive cause for the chylopericardium was identified. Given rapid pericardial effusion reaccumulation, and concern for the development of constrictive physiology secondary to chylopericarditis, the patient underwent combined thoracoscopic thoracic duct ligation and subtotal pericardiectomy via an open intercostal approach the following day. The lymphatic structures and thoracic duct were identified using near infrared fluorescent lymphography, as previously described (8). The thoracic duct was ligated with hemostatic clips (Allegiance Corporation, Missoula, MT). A subtotal pericardiectomy of the apical pericardium was performed ventral to the phenic nerves, leaving 1–2 cm of pericardium circumferentially around the base of the heart. A PleuralPort™ device (Norfolk Vet Products, Skokie, IL) was then placed as previously described (9).

Histopathology of the pericardium revealed moderate chronic multifocal, perivascular, lymphohistiocytic pericarditis with moderate to marked mesothelial hyperplasia and hypertrophy. No evidence of foreign material, infectious agents or neoplasia was observed.

The patient recovered for several days in hospital before being discharged on the following medications via the E-tube; maropitant 1.8 mg/kg SID, sodium bicarbonate 50 mg/kg BID, rivaroxaban 0.8 mg/kg BID, clopidogrel 1.8 mg/kg SID, sodium polystyrene sulfonate 0.3 g/kg TID, lanthanum carbonate 58 mg/kg TID, taurine 500 mg SID and carnitine 500 mg SID. The patient continued to return three times weekly for hemodialysis for two more months. No further pericardial effusion was present. Pleural effusion was consistently present, and the PleuralPort™ (Norfolk Vet Products, Skokie, IL) was used to remove 50–500 ml of pleural effusion 1-h before every hemodialysis treatment. The patient died at home of unknown cause 9 weeks following pericardiectomy. In total, the patient received 50 hemodialysis treatments over 4.5 months.

### Case 2

A 4-year-old male neutered 35 kg Doberman was referred for evaluation of progressive azotemia. On presentation, the dog was quiet, alert, and responsive with no major abnormalities on physical examination. Initial bloodwork revealed a mild normocytic, normochromic, non-regenerative anemia of 35.7% (RI 40%−55%). Biochemistry revealed a marked azotemia with serum creatinine of 11.5 mg/dl (RI: 0.8–1.5), BUN of 155 mg/dl (RI: 11–33), a metabolic acidosis (bicarbonate 13 mmol/L, RI: 20–29), marked hyperphosphatemia (15.9 mg/dl, RI: 2.6–5.2) and mild hypoalbuminemia (3.1 g/dl, RI: 3.4–4.3). A urine protein:creatinine ratio was mildly elevated at 1.5 (RI < 0.5) and bacterial urine culture and leptospirosis serology testing were negative. Abdominal ultrasonography revealed bilateral chronic degenerative renal changes suspected to be due to an underlying juvenile nephropathy. Hemodialysis was pursued for patient management.

A 14Fr × 24 cm double lumen silicone jugular catheter (MILA International, Inc, Florence, Kentucky) was placed aseptically into the right jugular vein without complication. A 30 Fr × 55 cm esophageal feeding tube (MILA International, Inc, Florence, Kentucky) was placed concurrently without complication. Thoracic radiographs confirmed appropriate dialysis catheter positioning, with termination in the cranial vena cava. The patient was hospitalized for 6 days and received two hemodialysis sessions using systemic heparin as anticoagulation during therapy. Following initial discharge from the hospital, the patient returned for twice weekly outpatient hemodialysis therapy and was receiving the following medications via the E-tube; amlodipine 0.18 mg/kg SID, ondansetron 0.5 mg/kg BID, omeprazole 0.6 mg/kg BID, clopidogrel 2 mg/kg, sodium bicarbonate 18 mg/kg TID, taurine 500 mg SID, carnitine 500 mg SID and aluminum hydroxide 48 mg/kg/day divided with each feeding. The catheter was heparin-locked (100–110 units/kg/ml UFH) between hemodialysis treatments. Darbepoetin was also given subcutaneously at 1 μg/kg weekly.

**Figure 4:**
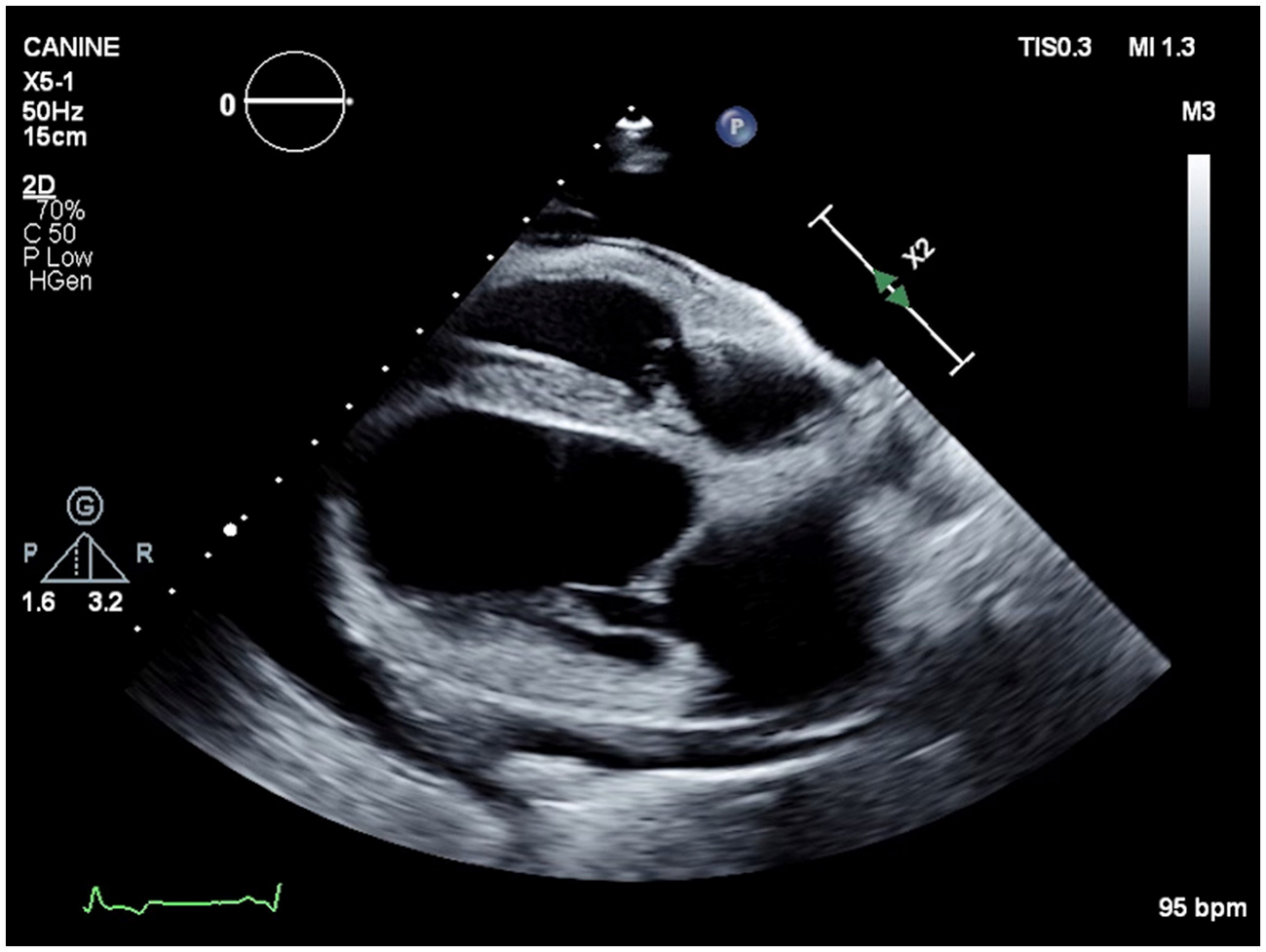
Echocardiographic image (right parasternal four chamber view) showing severe anechoic pleural effusion and mild anechoic pericardial effusion.

After 17 hemodialysis treatments, the patient presented for his scheduled hemodialysis therapy and increased respiratory effort was noted on physical examination. Thoracic ultrasonography revealed moderate pleural effusion. Echocardiogram showed scant volume of pericardial effusion with normal cardiac function (Figure 4). The patient's dialysis catheter was visualized coursing through the vena cava and terminating in the proximal right atrium. Suspected thrombi were noted associated with the portion of the catheter within the atrium. As the catheter was traced cranially during ultrasonographic examination, a large thrombus was visualized surrounding the catheter. Thoracocentesis was performed routinely with a 14G angiocatheter without complication and 2,100 ml of pink opaque pleural effusion was removed. Fluid analysis was consistent with a chylous effusion, with triglyceride content 600 mg/dl (serum triglycerides; 72 mg/dl). The patient was re-hospitalized and continued on medications as above, with the addition of rivaroxaban 0.8 mg/kg SID via the E-tube. Roughly 72 h after hospitalization, cervicothoracic ultrasonography confirmed a right jugular vein thrombus occluding an estimated 90% of the lumen. A 20G IV catheter was placed cranial to the jugular vein thrombus and sutured in placed with a T-port attached. A tissue plasminogen activator (TPA; Alteplase, Genentech, San Francisco, CA) constant rate infusion (CRI) of 0.5 mg/h was given via the jugular catheter over 12 h. The following morning, a repeat cervicothoracic ultrasound revealed moderate pleural and pericardial effusion. The jugular vein thrombus appeared static in size. A pericardiocentesis was performed using a 14G × 5.25 inch Angiocath™ catheter (BD, Edison, NJ) at the right 6th intercostal space after 1 ml of 2% lidocaine local block was administered. Roughly 150 ml of pink opaque fluid was removed. Fluid analysis was consistent with chylous effusion, with triglycerides 957 mg/dl (serum triglycerides; 146 mg/dl) and Sudan III positive staining. A thoracocentesis was subsequently performed as previously described, with 1,900 ml removed.

A thoracic CT scan and concurrent lymphangiogram was performed which confirmed right external jugular and cranial vena cava venous thrombosis associated with the patient's central venous dialysis catheter. Moderate pleural and pericardial effusion was also present.

A venogram was performed the following day under anesthesia, revealing significant narrowing of the lumens of the right external jugular vein and of the cranial vena cava. Under fluoroscopic guidance, a 0.035 mm angled hydrophilic guidewire was placed through the dialysis catheter and the catheter was removed over the guidewire. As the catheter was pulled, a large volume of adherent thrombus that was attached to the catheter shaft was also removed. An EN Snare^®^ Endovascular Snare System (Merit Medical) 12–20 mm variable diameter was then used to remove clot from the affected vessel. Following clot removal, the guidewire was replaced, and an 18G 1.3-inch catheter was placed over the guidewire and sutured into the proximal jugular vein. A CRI of TPA was instilled at 2.5 mg/h for 12 h through the jugular vein. The subsequent morning, a 0.035-inch guidewire was placed through the 18G catheter, the catheter removed and a new 11.5Fr × 24 cm dialysis catheter was placed. Hemodialysis was performed the same day. The patient was discharged to continue outpatient hemodialysis therapy three times weekly and received the following medications daily via the E-tube; amlodipine 0.26 mg/kg SID, clopidogrel 2 mg/kg, sodium bicarbonate 35 mg/kg TID, taurine 500 mg SID, carnitine 500 mg SID, sodium polystyrene sulfonate 0.3 g/kg TID, rivaroxaban 0.5 mg/kg BID, lanthanum carbonate 36 mg/kg TID and aluminum hydroxide 23 mg/kg TID. Owners declined moving forward with intrathoracic surgery, including thoracic duct ligation and/or pericardiectomy.

On the second hemodialysis session post-thrombus removal, recurrence of pericardial and pleural fluid was noted. Repeat pericardiocentesis and thoracocentesis were performed as described earlier, with removal of 300 and 1,700 ml respectfully of pink tinged white fluid. Fluid analysis was not repeated. The patient received hemodialysis and returned in 48 h for another hemodialysis session. Thoracic ultrasonography revealed recurrent pericardial and pleural effusion. Pericardiocentesis and thoracocentesis were repeated, removing 370 ml of pericardial fluid and 1,880 ml of pleural fluid. The patient received a standard hemodialysis treatment 1 h post fluid removal. Two hours after hemodialysis the patient collapsed. Ultrasound revealed progressive pericardial and pleural effusion despite removal earlier that day. The owners elected humane euthanasia. In total, the patient received 25 hemodialysis treatments over 2 months.

## Discussion

This is the first known report to document the development of chylopericardium in dogs undergoing chronic hemodialysis, suspected to be associated with long-term indwelling dialysis catheters and secondary catheter-associated thrombosis and/or stenosis. The development of chylopericardium secondary to thrombosis of the superior vena cava, jugular vein or subclavian vein is well documented in human patients (5, 6, 10, 11). Vena cava thrombosis and stenosis with subsequent chylopericardium have been reported in human patients with jugular catheters undergoing hemodialysis (12) and in human patients with central venous catheters in place (5, 13). Uremic pericarditis is documented in human patients with end-stage renal disease, suspected secondary to retention of uremic toxins although the underlying pathophysiology remains unknown (14, 15). Clinical consequences of uremic pericarditis include pericardial effusion, constrictive pericarditis, and pyrexia (14, 16, 17). While uremic pericarditis may potentially be an etiology for the chylous pericardial effusion in both cases presented, the finding of venous thrombosis large enough to cause venous hypertension and impaired lymphatic drainage made other causes of primary pericardial disease less likely. Histopathologic changes to the pericardium in case 1 were consistent with inflammation secondary to chyle (18), with no distinguishing primary cause of pericarditis observed.

Management of chylopericardium in humans includes both conservative and surgical approaches. Described conservative management include pericardiocentesis and fat restricted dietary change (1), with thrombolytic therapy and placement of self-expanding metallic stents (19). Dietary change was not undertaken in either case due to the dietary restrictions needed with end stage CKD. Both patients were fed a nutritionist formulated home-cooked diet.

In case 2, a less surgically invasive management approach was taken with thrombolytic therapy, attempted thrombectomy and catheter exchange. During catheter exchange, a large portion of the thrombus was removed attached and associated with the catheter. As the thrombus extended into the cranial vena cava, replacement of the catheter to the contralateral jugular vein would not have been helpful. In addition, given the extensive thrombus spread through the cranial vena cava, it is possible that thoracic duct ligation would not have resolved the effusion, as accessory ducts were likely to also be obstructed by the extensive clot. Venous angioplasty was not performed in either dog.

In case 1, pericardial effusion resolved after thoracic duct ligation and subtotal pericardiectomy, but pleural effusion persisted and required regular evacuation via the PleuralPort™ (Norfolk Vet Products, Skokie, IL). It is suspected in the case presented that the thoracic duct may have been incompletely ligated, or more likely that the persistent venous thrombus or venous stenosis prevented opening of collateral lymphatic vessels, resulting in persistent chylous pleural effusion as accessory ducts were also obstructed. The cause of death for case 1 was unknown, with cardiac arrythmias, hyperkalemia or thromboembolic event possible etiologies.

Both cases highlight complexities that can arise when managing a longer-term indwelling dialysis catheter in canine patients. A large central venous catheter is essential in veterinary patients undergoing hemodialysis. Catheter related thrombosis is the most common non-infectious related catheter complication in people with long-term indwelling venous catheters (20). In both cases in this report, a large thrombus formed around the dialysis catheter despite antiplatelet therapy (clopidogrel 1.5–2 mg/kg PO SID), locking of the catheter with high concentration heparin in the interdialytic period (100–110 units/kg/ml UFH) and systemic heparinization during hemodialysis two–three times weekly.

The pharmacokinetic and pharmacodynamic handling of antithrombotics in patients with advanced renal disease remains poorly understood, making dosing recommendations difficult. In human patients with end stage renal disease, rivaroxaban drug clearance decreases by 35% compared to healthy patients (21). Given this, initial dosing of antithrombotics for the oligoanuric patients in this report were decreased compared to published guidelines (22) to lessen the risk for spontaneous bleeding. Conversely, glomerular filtration rate (GFR) reduction in human patients has been associated with a decreased response to clopidogrel (23). This phenomenon has not been studied in dogs, however, may have played a role in clot formation in the dogs of this report, despite clopidogrel administration. More precise dosing regimens for clopidogrel and rivaroxaban in patients with CKD may be achieved by utilizing platelet function (24) and anti-Xa (25) assays, however neither are widely available. Monitoring rivaroxaban via prolongation of prothrombin time (PT) has also been proposed (25) however interindividual variability (25) and timing of sampling (26) make this test less reliable. Despite concerns for development of hemorrhage in the patients of this report, even with dose escalation of both rivaroxaban and clopidogrel, no excessive bleeding occurred even with pericardiocentesis and thoracocentesis.

The current report provides further insight into the etiology and management of chylopericardium in dogs. Chylopericardium should be considered as a rare consequence of prolonged central venous catheter placement. In addition, further studies to better elucidate changes in hemostasis and in antithrombotic drug metabolism and excretion in patients with azotemia are warranted.

## Data availability statement

The raw data supporting the conclusions of this article will be made available by the authors, without undue reservation.

## Ethics statement

Ethics review and approval and written informed consent were not required as per local legislation and institutional requirements.

## Author contributions

DW: Conceptualization, Investigation, Methodology, Writing – original draft, Writing – review & editing. CP: Conceptualization, Investigation, Methodology, Supervision, Writing – original draft, Writing – review & editing. LD: Conceptualization, Investigation, Methodology, Writing – original draft, Writing – review & editing. LC: Writing – review & editing, Writing – original draft. MA: Writing – review & editing, Writing – original draft. WC: Writing – review & editing, Writing – original draft.
